# Fostering Inductive and Deductive Learning in Oral Microbiology and Immunology With a Dual-Role Duel Card Game: Explanatory Sequential Mixed Methods Study

**DOI:** 10.2196/80048

**Published:** 2026-01-21

**Authors:** Kawin Sipiyaruk, Ratchapin Laovanitch Srisatjaluk, Ponpentip Chaisiri, Kanokwan Jantawatchai, Jidapha Srihatai, Chutikarn Sonyaem, Thanaphat Traikorakot, Suphasuta Pookboonmee

**Affiliations:** 1 Department of Orthodontics, Faculty of Dentistry, Mahidol University, 6 Yothi Road, Ratchathewi, Bangkok, Thailand, 66 2007813; 2 Mahidol International Dental School, Faculty of Dentistry, Mahidol University, Bangkok, Thailand; 3 Doctor of Dental Surgery Program, Faculty of Dentistry, Mahidol University, Bangkok, Thailand; 4 Medical Education Technology Center, Faculty of Medicine Siriraj Hospital, Mahidol University, Bangkok, Thailand

**Keywords:** active learning, card game, educational game, game-based learning, microbiology, immunology

## Abstract

**Background:**

Game-based learning has emerged as an effective learning strategy in health care education. However, no games have been specifically designed to support cognitive improvement for diverse learning styles in oral microbiology and immunology.

**Objective:**

This study aimed to develop and evaluate an educational card game designed to support diverse learning styles in oral microbiology and immunology, using a duel-style format.

**Methods:**

An explanatory sequential mixed methods study was conducted with 40 third-year dental students, where half of them were assigned to the first group, starting as the host, while those in the other group began as the microbe. Participants alternated between the microbe and host roles during gameplay. Active engagement through playing as the microbe facilitated knowledge acquisition through observation, supporting inductive learning. On the other hand, the host role aimed to promote the application of knowledge for decision-making, facilitating deductive learning. Quantitative data were collected using pre- and postknowledge assessments and satisfaction questionnaires. Qualitative insights were obtained through semistructured interviews exploring learning experiences when playing as the microbe compared to the host.

**Results:**

Students demonstrated significant improvements in knowledge scores across the 3 assessments (*P*<.001), with no difference between groups (*P*=.85). They also perceived the game positively in all 3 aspects (usefulness, ease of use, and enjoyment). Qualitative findings revealed that role variation supported both inductive and deductive learning processes. Participants valued the combination of pedagogical and entertaining components, leading to the game’s motivation and engagement. A conceptual framework demonstrated key emerging themes relevant to the game design and implementation, including learner profile, learning setting, game design, learning process, and learning outcomes.

**Conclusions:**

The card game demonstrated its potential in enhancing knowledge acquisition and student engagement in oral microbiology and immunology. Role-switching between the host and microbe was perceived by participants to facilitate different learning experiences. Further research is recommended to investigate long-term retention and broader practicality.

## Introduction

Oral microbiology provides essential insights into bacteria, viruses, and fungi in the oral cavity, while immunology focuses on the mechanisms by which the human body defends itself against these pathogens. This knowledge enables health care professionals, including dental practitioners, to understand pathogen transmission, immune interactions, and strategies for disease management and prevention [1-3]. The COVID-19 pandemic, for example, significantly impacted dental practices by introducing new protocols to reduce viral transmission through respiratory droplets [4,5]. Studying microbiology and immunology is therefore important for understanding infectious diseases, and it has proven essential for health care education.

Traditional teaching methods have served as the main method of education worldwide. While this approach can reduce anxiety by minimizing the need for active student participation, it can limit engagement due to the predominantly 1-way communication [6,7]. Disengagement in traditional classrooms has been shown to negatively impact academic performance [8-10]. On the other hand, active learning strategies, which promote critical thinking and student interaction, are associated with better engagement [11,12]. In addition, not all students have the same learning preferences [13]. Some of them prefer direct participation and decision-making, while others may learn more effectively by observing patterns. Educational tools designed for both learning preferences may support diverse learning needs.

Game-based learning has been considered an innovative strategy to address the limitations of traditional teaching methods by incorporating game mechanics into educational settings. Game-based learning has been applied in medical and dental education [14-17], including microbiology and immunology [18,19]. This approach is widely recognized due to its ability to enhance student engagement and motivation in various disciplines of dental education, such as dental public health [20], human anatomy [21], oral diagnosis [22,23], orthodontics [24], endodontics [25], patient safety [26], clinical administration [27], and teledentistry [28]. These games can be implemented through various formats, ranging from digital simulations to physical formats such as board and card games.

Despite the growing number of educational games, most of them are not designed to accommodate a range of learning preferences or gameplay experience levels. In other words, they may not explicitly support both inductive (structured observation) and deductive learning (active problem-solving), which are 2 distinct approaches through which students engage with and make sense of content. This study aimed to develop and evaluate an educational card game designed to support diverse learning needs in oral microbiology and immunology, using a duel-style format in which players alternate between the roles of microbe and host to simulate infection and immune response dynamics. Specifically, the objectives were to assess the game’s effectiveness in improving knowledge acquisition and to investigate how learners engaged differently when beginning as the microbe compared to the host.

## Methods

### Game Development

#### Game Concept

The “Invasion” game was designed to support teaching and learning in oral microbiology and immunology, where players alternate roles between microbes and hosts. This game served as a novel educational tool to engage dental students more deeply with the learning content. The game was designed to align with the expected learning outcomes in oral microbiology and immunology, requiring learners to apply their knowledge of microbial pathogenesis and human immune responses to infection. The role alternation within the game provides different forms of engagement, encouraging learners to interact with the content either through structured observation as microbes (inductive learning) or through direct decision-making as hosts (deductive learning). A physical card format was chosen because it encourages face-to-face interaction, peer discussion, and active participation, which strengthens the learning experience when students switch between roles. In addition, compared with a digital format, this design helps students remain focused on the learning activity itself rather than being distracted by technological navigation.

#### Instructional Design and Learning Tasks

The pedagogical framework of this game is based on the constructivist learning theory, where learners are expected to actively construct new knowledge through problem-solving and critical reflection, based on their existing understanding. Engagement with the game elements can allow students to develop a deeper understanding of microbiological and immunological concepts. The dual-role format supports different types of cognitive engagement. Playing as the microbe emphasizes reflective observation and the construction of clues (inductive learning), while the host role requires active problem-solving and the application of knowledge to identify pathogens and apply appropriate responses (deductive learning). Another important component of the game is the feedback mechanism. Each action taken by the player provided an opportunity for formative assessment, allowing for real-time correction and learning from mistakes. The feedback mechanism can support learners to adjust their strategies and understandings during the gameplay. Through this process of testing, feedback, and adjustment, students can engage in a learning cycle that mirrors scientific reasoning and clinical decision-making.

#### Game Interface and Interaction

**Figure 1.**
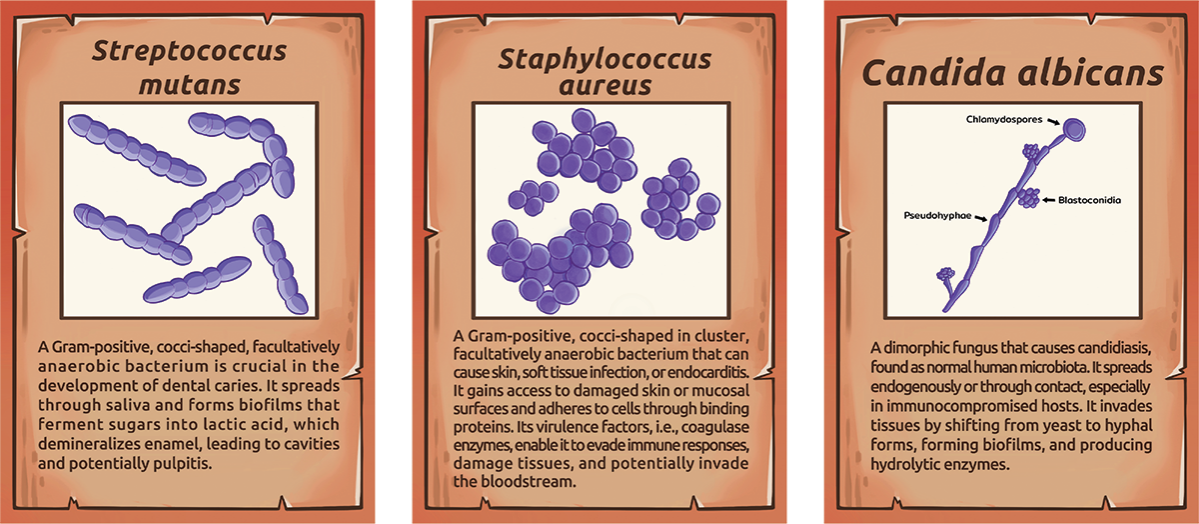
Examples of “Microbe” cards used in the “Invasion” game.

According to the gameplay mechanics, the game begins with the microbe player selecting 3 “Microbe” cards, along with their corresponding “Key” cards and 7 “Hint” cards (Figure 1). The host, in turn, starts the game with 5 heart points and a set of 7 “Protection” cards. During gameplay, the microbe presents “Hint” cards, prompting the host to select 1 of 3 actions: play up to 3 “Protection” cards in response to the clues, discard 3 “Protection” cards in exchange for a “Key” card offering more detailed information, or use an “Attack” card to guess the identity of a microbe. A correct guess leads to the revelation of that microbe, while an incorrect guess results in a loss of heart points calculated as the number of “Hint” cards played minus the number of correctly placed “Protection” cards. Game completion occurs when the host successfully identifies 3 microbes, resulting in a host victory, or when all heart points or “Attack” cards are used, which means the microbe player wins.

**Figure 2.**
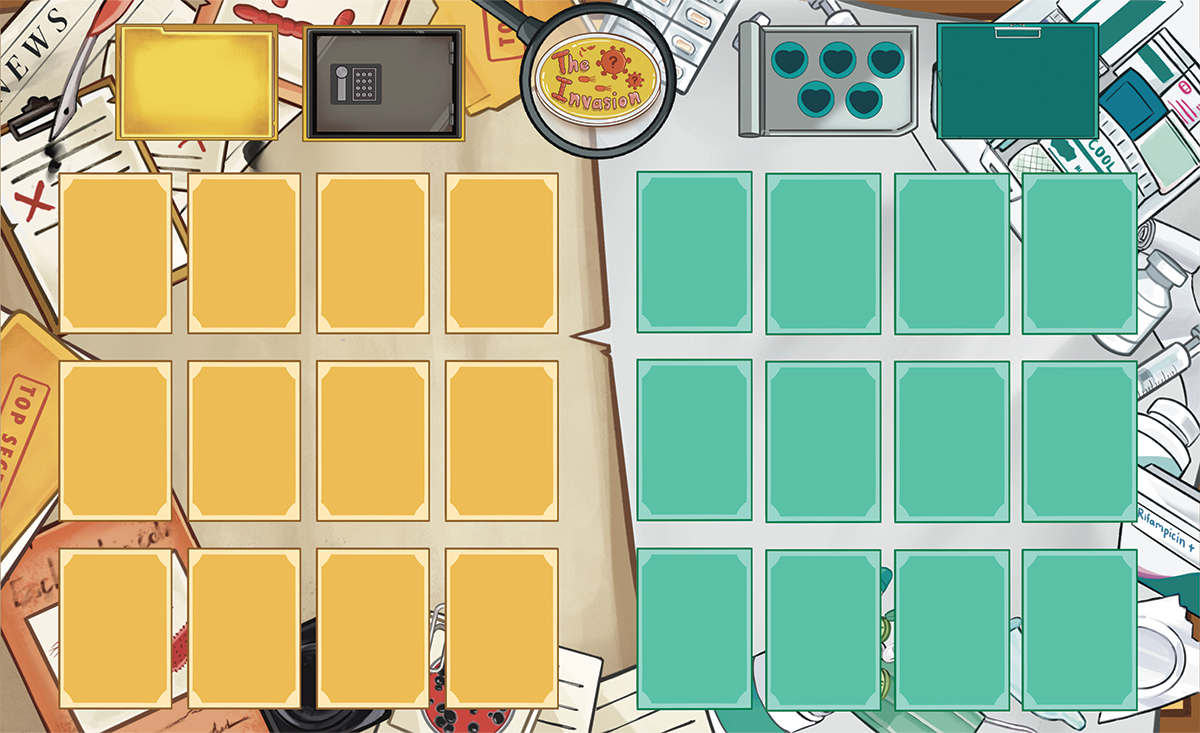
The cardboard with designated spaces for card placement in the “Invasion” game.

To further support gameplay, a cardboard was designed as an organizing component (Figure 2). The cardboard provided designated spaces for different types of cards (eg, microbe, host, or support actions), guiding players in organizing card placement throughout the game. It was intended to reduce confusion during gameplay, thereby enhancing usability and the overall learning experience. Its purpose was to minimize the cognitive load related to card management, allowing players to focus more on the learning tasks.

Effective gameplay requires the microbe player to present hints in a way that is strategically challenging to test the host’s interpretation skills. In turn, the host must analyze the clues, make careful decisions about resource use, and minimize incorrect guesses in order to preserve their heart points. These interactive dynamics create a learning environment that reflects the cognitive and analytical challenges involved in diagnosing and managing microbial infections in clinical practice. By placing students in alternating roles with distinct cognitive demands, the game supports the development of microbiological understanding, analytical thinking, and adaptability, which are skills essential for clinical reasoning and for meeting the varied learning needs of students. This gameplay structure is designed to engage students in different types of thinking, depending on their assigned role. A summary of the game components is provided in Table 1.

**Table 1.** Description of card types used in the game.

Card type	Used by	Description
Microbe cards	Microbe	Represent various microbes selected for their clinical relevance in dentistry. These cards define the core challenges posed by the microbe player during gameplay.
Protection cards	Host	Provide the host with strategies to prevent or reduce damage from incorrect microbial identification. Each card contains information on treatments, immune responses, or behavioral changes.
Hint cards	Microbe	Contain clues related to the microbe’s characteristics, such as virulence factors, clinical manifestations, and transmission routes. They support progressive disclosure of information to the host.
Key cards	Microbe	Offer more specific and detailed information about a microbe than “Hint” cards, enhancing the host’s ability to make accurate identifications. Obtained through card exchange.
Attack cards	Host	Allow the host to make an informed guess about the identity of a microbe based on accumulated clues. Limited to 5 per game, requiring careful use.

### Research Design

This study used an explanatory mixed methods design to evaluate the “Invasion” game and to examine differences in learning experiences and outcomes when students engaged in gameplay as either a microbe or a host.

The quantitative phase used an experimental design with partial randomization to assess knowledge acquisition and compare learning outcomes based on gameplay role order. A total of 40 participants were allowed to self-select their partners, forming pairs based on personal preference to reflect natural peer interactions, commonly found in educational settings [21]. Within each pair, roles were randomly assigned using a coin toss method to determine which player would begin as the host or the microbe. Prior to gameplay, participants completed a preknowledge test (Pretest), followed by 1 round of the “Invasion” game in their assigned role. A postknowledge test (Posttest 1) was administered immediately after the first round. In the second round, participants switched roles and completed another postknowledge test (Posttest 2). A satisfaction questionnaire was also completed at the end of the second round. All test items, game materials, and instructions were provided in English.

For the qualitative phase, all participants took part in short semistructured interviews to explore their experiences and learning processes while engaging with both gameplay roles. This approach provided clarification on how the game’s dual-role structure facilitated distinct forms of engagement, with students tending to adopt a more reflective and observational approach when playing as the microbe, and a more analytical and decision-driven approach when playing as the host. All 40 participants were interviewed to ensure full representation of perspectives across both gameplay roles and to enhance the integration of qualitative insights with quantitative findings. This decision was made because the approach was intended not only to achieve thematic saturation but also to ensure that the qualitative data contextualized the quantitative results, while the brief and focused nature of the interviews made it feasible to include all participants. The semistructured format allowed for a consistent set of guiding questions while providing flexibility to probe deeper into participant reflections. Interviews were conducted immediately after the quantitative phase to ensure relevance and recall.

**Figure 3.**
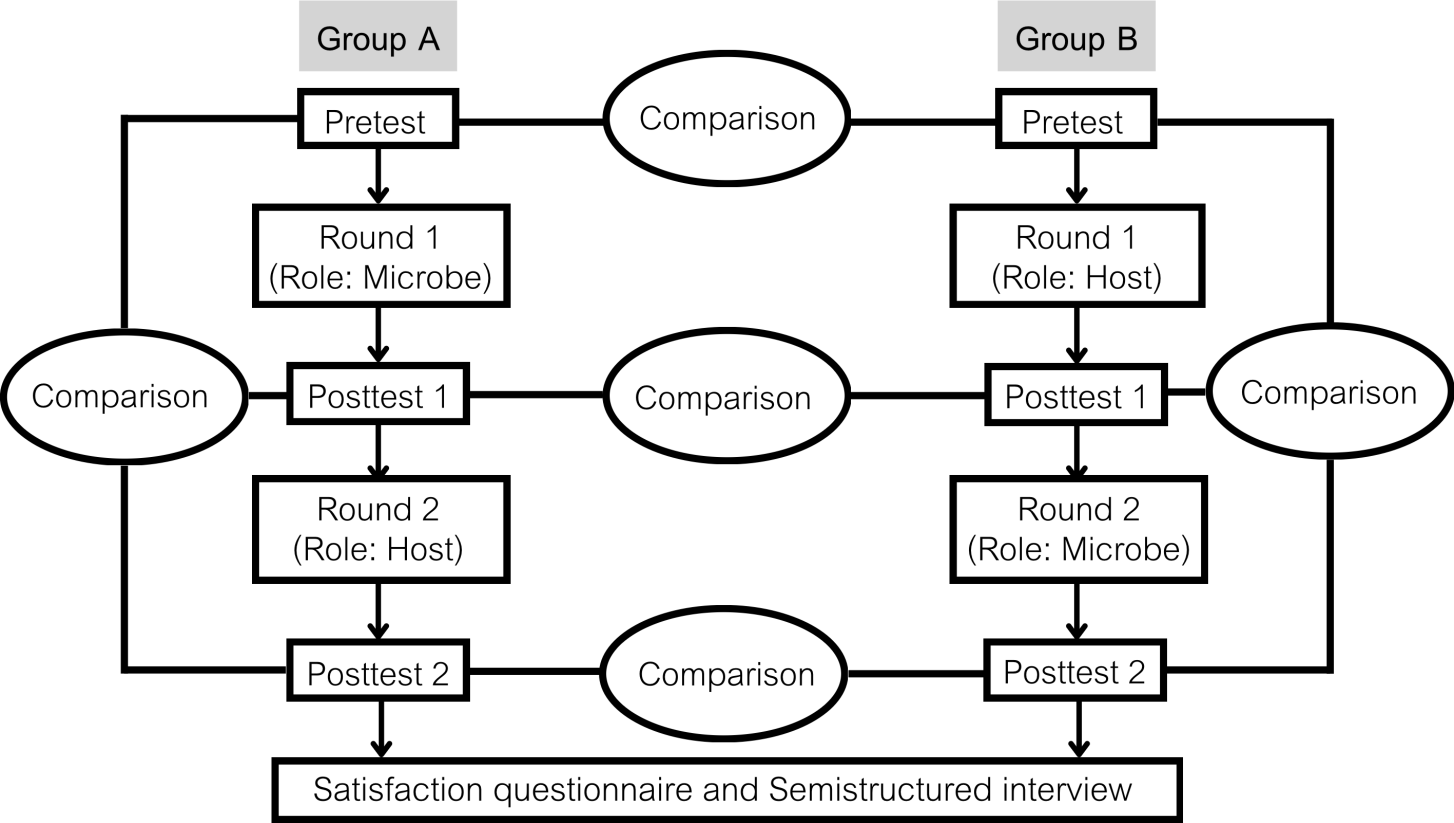
Diagram presenting the outline of the study design.

All procedures were completed in a single session, where each game round (host or microbe) lasted approximately 25‐30 minutes. All participants completed their activities under the same conditions, and knowledge assessments were completed independently within a fixed time frame. This standardized approach ensured consistency and comparability of learning experiences across groups. The full study protocol is illustrated in Figure 3.

### Research Participants

The research population consisted of third-year undergraduate dental students enrolled in the 2024 academic year at the Faculty of Dentistry, Mahidol University. They were eligible if they had successfully completed 3 prerequisite courses: Fundamentals of Medical Microbiology for Dental Students, Essential Immunology for Dental Students, and Oral Microbiology and Immunology. Completion of these courses ensured a baseline understanding of core concepts relevant to the game content. While all game materials and knowledge assessments were designed in English, students with insufficient English proficiency to understand the game instructions were excluded from the study to ensure that language barriers did not affect gameplay engagement or performance. This exclusion was determined through an orientation session in which students were asked to demonstrate comprehension of the English instructions. Additionally, any participant who failed to complete a required task within the allocated time was withdrawn.

The required sample size was determined using G*Power software (version 3.1; Heinrich-Heine-Universität Düsseldorf). According to a previous study [21], the mean and SD of the pretest score were 9.37 and 2.40, respectively, while those of the posttest score were 11.37 and 1.96. With a statistical power of 95% and a significance level of *P*=.85, the required sample size per group was 18, resulting in a minimum total of 36 participants. To compensate for a potential 10% dropout rate, the sample size was set at 40 participants (20 per group).

Participant allocation followed a 2-step process. Students first formed pairs through voluntary matching. Within each pair, a coin toss was used to randomly assign 1 participant to begin as the host and the other as the microbe. This randomization step helped mitigate potential bias introduced by voluntary pairing. Allowing students to choose their own partners made the activity feel more natural, while randomly assigning gameplay roles ensured a fair comparison between the host and microbe roles. By assigning roles randomly, the study preserved experimental control and enabled the qualitative phase to capture the experiences of participants who may have been placed in roles misaligned with their learning preferences. This role mismatch provided valuable insight into the cognitive and emotional challenges encountered by different learners, offering a more comprehensive understanding of the game’s effectiveness and highlighting both the strengths and limitations of its design across diverse learner profiles.

### Data Collection Tools

#### Knowledge Assessments

To evaluate knowledge acquisition, 3 structured assessments were administered at different stages of the study: Pretest, Posttest 1, and Posttest 2. Pretest aimed to assess students’ baseline understanding and help identify individual learning gaps prior to the intervention. Posttest 1 and Posttest 2 were used to measure knowledge gains following participation in the learning activities. Each assessment consisted of the same 20 multiple-choice questions to ensure consistency in content and difficulty. However, to minimize the influence of recall bias, both the order of the questions and the arrangement of answer choices were randomized across the assessments.

Content validity was confirmed through expert review by 3 microbiologists who were not involved in the study. These experts evaluated each question for its relevance and alignment with the expected learning outcomes. The Index of Item-Objective Congruence (IOC) was used, with items scoring below 0.5 being revised based on expert feedback. Face validity was assessed by a group of fourth-year dental students who had completed the same microbiology and immunology courses required for inclusion in the study. Involving these senior students helped ensure that the test items were appropriate and clearly worded, and this approach prevented contamination of the research participants.

#### Satisfaction Questionnaire

To investigate user perceptions of the learning experience, a self-administered questionnaire comprising 18 items was used. Each item was rated on a 5-point Likert scale, ranging from 1 (“Strongly disagree”) to 5 (“Strongly agree”), and adapted from previously validated instruments [22,27,28]. The questionnaire assessed 3 key dimensions commonly used in the evaluation of serious games: perceived usefulness, ease of use, and enjoyment. Content validity was established through expert review by 3 dental educators, who evaluated each item for relevance and alignment with the intended constructs. Items with an IOC below 0.5 were revised through iterative feedback until acceptable agreement was achieved.

#### Semistructured Interviews

To complement the quantitative findings on how different gameplay roles supported varied learning approaches, semistructured interviews were conducted using a topic guide developed by the research team. The topic guide comprised 5 main areas, with flexibility to probe further based on participants’ responses. These questions examined students’ reflections on the learning experience, the influence of assigned roles (microbe or host) on learning and engagement, and suggestions for improvement. Interview responses were audio-recorded with permission and transcribed using a verbatim technique. This process ensured that the learning experiences were captured with depth and fidelity.

### Data Analysis

Quantitative data were analyzed using IBM SPSS Statistics for Windows, version 29.0. Descriptive statistics were used to summarize participant characteristics and assessment scores. A 2-way repeated-measures ANOVA was conducted to examine the effects of assigned role order (host and microbe) and time (Pretest, Posttest 1, and Posttest 2). Bonferroni-adjusted post hoc tests were used to explore pairwise differences across time and between groups. Assumptions of normality and sphericity were verified prior to analysis to ensure that the requirements for repeated-measures ANOVA were met. Data from the satisfaction questionnaire were analyzed using means and SDs. Statistical significance was set at *P*<.05.

Qualitative data were analyzed using thematic analysis. Two researchers independently reviewed the transcripts to identify recurring patterns, followed by discussion and consolidation of themes with the corresponding author. The analysis specifically examined how the 2 gameplay roles (host and microbe) contributed to learners’ knowledge improvement. This focus aligned with the research objective of supporting diverse learner profiles through role variation. Credibility, consistency, and transparency were prioritized throughout the analytic process to ensure the rigor of interpretation.

### Ethical Considerations

This study received ethical approval from the Institutional Review Board of the Faculty of Dentistry and the Faculty of Pharmacy, Mahidol University (MU-MOU CoA 2025/013.1002). Before data collection, all participants were provided with a detailed information sheet and given written informed consent. To protect confidentiality, all data were anonymized, and no personally identifiable information was available during analysis. No compensation was provided to participants.

## Results

### Research Participants

A total of 40 third-year undergraduate dental students participated in the study. Participants were randomly assigned to 2 groups of 20 students each. Group A consisted of students who began as the microbe (6 male and 14 female), while Group B included those who started as the host (3 male and 17 female). All participants completed the assigned tasks, and no dropouts occurred during the study.

### Knowledge Assessments

Participants in both groups demonstrated progressive improvements in knowledge scores across the 3 assessments (Table 2). For Group A, the mean score increased from 9.85 (SD 3.72) at Pretest to 12.4 (SD 3.65) at Posttest 1, and further to 14.55 (SD 3.38) at Posttest 2. Similarly, Group B improved from 9.55 (SD 2.44) at Pretest to 11.8 (SD 3.07) at Posttest 1, and then to 15.9 (SD 2.05) at Posttest 2. These results suggest that both groups experienced consistent knowledge gains following each round of gameplay.

**Table 2.** Knowledge assessments across time points by group.

Group	Assessment	Mean score (SD)	95% CI
A	Pretest	9.85 (3.72)	8.43 to 11.27
	Posttest 1	12.4 (3.65)	10.87 to 13.93
	Posttest 2	14.55 (3.38)	13.29 to 15.82
B	Pretest	9.55 (2.44)	8.13 to 10.97
	Posttest 1	11.8 (3.07)	10.27 to 13.33
	Posttest 2	15.9 (2.05)	14.64 to 17.17

### Effects of Time and Group (A 2-Way Repeated-Measures ANOVA)

A 2-way repeated-measures ANOVA was conducted to examine improvements in knowledge scores over time of assessments and whether there were differences between the 2 groups (host-first vs microbe-first). According to Table 3, the analysis demonstrated a significant main effect of time (*F*_2,76_=54.02; *P*<.001), indicating that there were improvements in knowledge scores across the 3 assessments. However, the interaction between time and group was not statistically significant (*F*_2,76_=1.94; *P*=.15), suggesting that both groups demonstrated a similar pattern of improvement over time. The between-subjects analysis showed no significant main effect of group on knowledge scores (*F*_1,38_=0.038; *P*=.85), indicating that initial group assignment did not influence overall performance (Table 4).

**Table 3.** Results of 2-way repeated-measures ANOVA examining within-subjects effects.

Source	Type III sum of squares	Mean square	*F* test (*df*)	*P* value	$\eta_{p}^{2}$
Time	614.017	307.008	54.02 (2, 76)	<.001	.587
Time × group	22.050	11.025	1.94 (2, 76)	.15	.049
Error (time)	431.933	5.683	—^a^	—	—

a Not applicable.

**Table 4.** Results of 2-way repeated-measures ANOVA examining between-subjects effects.

Source	Type III sum of squares	Mean square	*F* test (*df*)	*P* value	$\eta_{p}^{2}$
Group	0.675	0.675	0.04 (1, 38)	.85	.001
Error (group)	672.317	17.693	—^a^	—	—

a Not applicable.

### Pairwise Comparisons of Assessment Time Points

Given the absence of a significant interaction between time and group and no main effect of group, Bonferroni-adjusted pairwise comparisons were conducted by combining data from both groups. As shown in Table 5, knowledge scores increased significantly between each assessment point: from Pretest to Posttest 1 (*P*<.001), from Posttest 1 to Posttest 2 (*P*<.001), and from Pretest to Posttest 2 (*P*<.001). These findings reflect a cumulative learning effect over time. To further explore learning progression within each role-assigned group, Bonferroni-adjusted pairwise comparisons were performed separately within each group (Table 6).

**Table 5.** Bonferroni-adjusted pairwise comparisons of knowledge scores across assessment time points.

Comparison		Mean difference (95% CI)	SE	*P* value
Pretest	Posttest 1	−2.4 (−3.696 to −1.104)	0.517	<.001
Pretest	Posttest 2	−5.525 (−6.889 to −4.161)	0.545	<.001
Posttest 1	Posttest 2	−3.125 (−4.47 to −1.78)	0.537	<.001

**Table 6.** Bonferroni-adjusted pairwise comparisons of knowledge scores across assessment time points within each group.

Group	Comparison		Mean difference (95% CI)	SE	*P* value
A	Pretest	Posttest 1	−2.55 (−4.382 to −0.718)	0.732	.004
	Pretest	Posttest 2	−4.70 (−6.629 to −2.771)	0.770	<.001
	Posttest 1	Posttest 2	−2.15 (−4.052 to −0.248)	0.759	.02
B	Pretest	Posttest 1	−2.25 (−4.082 to −0.418)	0.732	.01
	Pretest	Posttest 2	−6.35 (−8.279 to −4.421)	0.770	<.001
	Posttest 1	Posttest 2	−4.10 (−6.002 to −2.198)	0.759	<.001

### User Satisfaction

Participants from both groups perceived the game as a positive experience in all aspects: perceived usefulness, ease of use, and enjoyment (Table 7). Although Group B demonstrated slightly higher scores across all categories, there were no statistically significant differences between the groups (*P*>.05).

**Table 7.** Comparison of user satisfaction between the 2 groups.

Perceptions	Group A, mean (SD)	Group B, mean (SD)	Mean difference (95% CI)	*P* value
Perceived usefulness	4.51 (0.50)	4.53 (0.40)	0.02 (−0.31 to 0.27)	.91
Perceived ease of use	4.58 (0.51)	4.64 (0.34)	0.07 (−0.34 to 0.21)	.63
Perceived enjoyment	4.63 (0.55)	4.67 (0.55)	0.03 (−0.39 to 0.32)	.85

### Learner Experiences When Engaging Within the Gameplay

Semistructured interviews were conducted to gain in-depth information about the gameplay experiences of participants, exploring how their initial roles (host-first or microbe-first) influenced their learning engagement and perceptions of the game. The following key themes emerged from the thematic analysis, highlighting factors that shaped learners’ experiences during gameplay.

#### Theme 1: Learner Profile

##### Background Knowledge

Participants reported that their background knowledge significantly influenced their engagement and effectiveness during gameplay. Several students described challenges when their prior understanding was limited, particularly when they were required to make decisions. To manage these difficulties, participants strategically chose when to read detailed card descriptions, focusing more deeply on unfamiliar or difficult topics.

I read the description on the ‘Microbe’ and ‘Hint’ cards in detail only if I had limited knowledge about that card. If I was familiar with that microbe, I just read it briefly and sometimes just the heading.

[Participant 15, Group A, Female]

When I was the host, it was challenging to identify the microbes, because my microbiology knowledge wasn't that strong. Imagining just one microbe out of many possibilities felt difficult.

[Participant 32, Group B, Male]

##### Personal Preferences

Personal preferences toward the game can be considered as having a key role in shaping their engagement and emotional responses. Preferences for specific roles were likely to be influenced by individual perceptions of stress, decision-making enjoyment, and familiarity with game-based learning. Those who preferred less cognitive demand tended to favor the microbe role, while others were more motivated by the host role’s active decision-making component. Their preference for card games also contributed to positive attitudes, enhancing their overall willingness to engage and learn.

I prefer learning in a more relaxed setting, so playing as the microbe suited me, as it didn’t require as much intense thinking.

[Participant 12, Group A, Male]

I enjoy making decisions and being in control, so the host role felt more engaging and aligned with how I like to learn.

[Participant 31, Group B, Female]

I’ve always loved card games, so learning microbiology and immunology through one just made sense to me. It didn’t feel like studying, as I was having fun, but I could tell I was actually learning something useful at the same time.

[Participant 32, Group B, Male]

### Theme 2: Learning Process

Participants demonstrated differing patterns of engagement depending on whether they began the game as the microbe or the host, with each sequence influencing how they processed and applied microbiology and immunology content.

#### Host Before Microbe

Participants who began as the host described the experience as cognitively demanding but motivating. Without access to structured content at the outset, learners were required to draw on prior knowledge, make inferences, and formulate hypotheses, which align with higher-order thinking. This initial challenge appeared to increase engagement and stimulate curiosity.

I liked being the host first—it was challenging because I had no idea which microbes were involved. I had to gather information from both sides and think it through before attacking.

[Participant 32, Group B, Male]

Guessing what the microbe was felt exciting. I was genuinely happy when I got it.

[Participant 28, Group B, Female]

In subsequent rounds, these learners reported reflecting on their earlier performance, identifying knowledge gaps, and actively revisiting content to improve.

When I became the microbe, I went back and reviewed the Matching Key to see if I had used the right ‘Protection’ card before. I also wanted to know what other protections were possible because I had made mistakes in the last round.

[Participant 36, Group B, Female]

#### Microbe Before Host

Students who started as the microbe described this role as a preparatory stage, providing them with a broad overview of key concepts. This role encouraged exposure to content in a lower-pressure context, which later supported more confident and efficient decision-making when switching to the host role. The structure of the game allowed them to gradually transition from recognition-based learning to recall and application.

Playing as the microbe first gave me an advantage. It helped me remember the content for each microbe. So, when I switched to the host role, I could recall those features more quickly.

[Participant 15, Group A, Female]

Some participants also reported using short-term memorization techniques during the microbe phase, which helped build a basic conceptual scaffold for identifying pathogens in the host round. These findings suggest that beginning as the microbe may support initial content familiarization, enabling learners to make more informed and confident decisions when transitioning to the more cognitively demanding host role.

Since I started as the microbe, I tried to memorize the Matching Key. It helped me get a rough idea of which microbes stood out, so when I switched to host, I could answer based on what I remembered from the last round.

[Participant 5, Group A, Female]

### Theme 3: Game Design

The design of the game played an important role in shaping both the cognitive and emotional aspects of learning. Participants reflected on how the game’s structure, feedback mechanisms, visuals, and interactive elements supported their engagement and understanding.

#### Pedagogical Components

##### Learning Content

Participants reported they could learn about microbes and immunology from the information on the cards, whether by reviewing their own cards or observing those played by their opponents. They also noted that repeatedly experiencing the cards during gameplay deepened their understanding and helped them retain key concepts.

The cards had clear explanations. Sometimes I learned more from looking at my opponent’s cards.

[Participant 14, Group A, Female]

##### Level of Difficulty

Participants generally perceived the level of difficulty as appropriate for their academic background. The content was challenging enough to stimulate interest, but it was not too complex to create frustration. This balance helped sustain motivation throughout gameplay. However, some noted that repeated play led to familiarity with answers, reducing difficulty over time.

The content was just the right level of difficulty; it wasn’t too hard or too easy.

[Participant 8, Group A, Female]

The game was fun, but when I started to remember the answers, it became easier, so I’d love to see more variety in the microbes to keep it challenging.

[Participant 36, Group B, Female]

##### Feedback Mechanism

The review phase where players checked their “Protection” cards against the correct answers was consistently mentioned as a key learning moment. This immediate feedback helped participants reflect on their decisions, correct misunderstandings, and reinforce correct associations between microbes and immune responses.

I learned a lot from checking whether the Host’s ‘Protection’ card matched the microbe. Even if it was wrong, I still got to learn from that mistake.

[Participant 13, Group A, Male]

### Entertaining Components

#### Visual Engagement

The visual design of the game, including colorful illustrations and a playful layout, added to its appeal and enhanced the learning atmosphere. Many participants described the cards as cute or attractive, which positively influenced their willingness to engage.

The game design was really well done. The pictures were cute and made the whole experience more inviting.

[Participant 21, Group B, Female]

#### Active Decision-Making Elements

Gameplay was structured to allow active involvement from both players. While the host engaged in decision-making, the microbe observed and predicted responses. This dynamic encouraged deeper analysis and reflection on the game’s logic and strategy.

Watching my friend play gave me time to see how the game worked overall. I was thinking about the clues they used and whether the microbe I had in mind matched what they played.

[Participant 4, Group A, Female]

#### Social Interaction

Interactions between players, including discussion, guessing, and bluffing, contributed to enjoyment and emotional investment. Participants appreciated the playful tension between roles, which made learning more immersive and memorable.

I liked playing as the microbe because I could watch my friend guess and have fun trying to throw them off with my clues. I didn’t enjoy guessing as much, but I loved being the one giving hints.

[Participant 10, Group A, Female]

### Theme 4: Learning Setting

The physical and contextual conditions under which gameplay occurred played a notable role in shaping students’ comfort, focus, and willingness to engage with the learning material. Participants appreciated the autonomy offered by the flexible setting and timing, as well as the availability of technical support when needed.

#### Learning Atmosphere

Allowing participants to choose their preferred learning environment fostered a greater sense of comfort and autonomy. Many students reported feeling less anxious and more willing to take intellectual risks when playing outside a formal classroom setting. The absence of an authoritative figure reduced pressure and created space for experimentation and open guessing.

I liked that we could choose where to play … even if I wasn’t sure about an answer, I felt okay taking a guess without worrying that someone would judge me for getting it wrong.

[Participant 22, Group B, Male]

#### Flexibility in Learning Schedule

The flexibility to decide when to engage with the game contributed to a more focused and self-directed learning experience. Participants appreciated that they could play when they felt most ready, which made learning feel more like a voluntary activity than a scheduled obligation.

The game didn’t force us to play at a specific time. I could just pick it up whenever I wanted, and that made it feel like something I chose to do … not something I was made to do.

[Participant 15, Group A, Female]

#### Real-Time Gameplay support

Access to facilitators or staff members during gameplay reassured participants and minimized concerns about game mechanics. This allowed them to devote more attention to the educational content rather than the rules. The ability to clarify doubts in real-time improved confidence and supported sustained engagement.

Having staff there to answer questions made me less worried about the rules, so I could actually focus on the content and not get stuck on how to play.

[Participant 27, Group B, Female]

### Theme 5: Learning Outcomes

Gameplay provided opportunities for cognitive engagement across multiple dimensions, from foundational knowledge acquisition to higher-order reasoning (Table 8). Participants described improvements in knowledge, critical and strategic thinking, and conceptual understanding, highlighting the game’s educational value beyond passive content delivery.

**Table 8.** Frequency of reported learning outcomes achieved from the game.

Learning outcomes	Frequency
Knowledge recall	40
Knowledge acquisition	34
Critical thinking	30
Strategic thinking	26
Knowledge application	22

#### Knowledge Recall

As students alternated roles and revisited familiar content, their ability to retrieve specific microbial characteristics improved. The act of seeing clues repeatedly triggered memory recognition, reinforcing their confidence and retention.

Seeing the clues helped trigger my memory when I looked at the ‘Key’ card, the name of the microbe felt familiar. And when it was revealed, it confirmed that I remembered it right.

[Participant 22, Group B, Male]

#### Knowledge Acquisition

The game facilitated the acquisition of microbiology and immunology knowledge through its structured and interactive format. Repeated exposure to microbial features and host responses during gameplay helped build a stronger conceptual foundation. Many participants reported gaining new information simply by observing their opponent’s moves.

Reading the details on the cards played by my opponent gave me new information and helped me figure out the microbe.

[Participant 8, Group A, Female]

#### Critical Thinking

Participants engaged in critical thinking by interpreting incomplete information and making evidence-based guesses. Particularly in the host role, students described the need to synthesize clues and evaluate possible answers, moving beyond memorization to diagnostic reasoning.

When I played as the host, I had no idea what the microbe was at first. I had to slowly piece things together using the information I had.

[Participant 17, Group A, Male]

#### Strategic Thinking

The game structure encouraged participants to develop and adjust strategies based on observations of their opponent. This adaptive thinking extended beyond factual recall to prediction, planning, and real-time decision-making.

My opponent rarely played ‘Protection’ cards. He used a different strategy, like discarding cards to draw a keyword because it gave them a better advantage.

[Participant 18, Group A, Female]

#### Knowledge Application

Participants not only recalled learning content but also applied their knowledge during gameplay. They demonstrated an ability to connect microbiological information to real-life decision-making, such as selecting appropriate treatments or interventions.

I learned which medications go with which types of microbes.

[Participant 9, Group A, Female]

### Conceptual Framework for the Implementation of the Game in Dental Education

**Figure 4.**
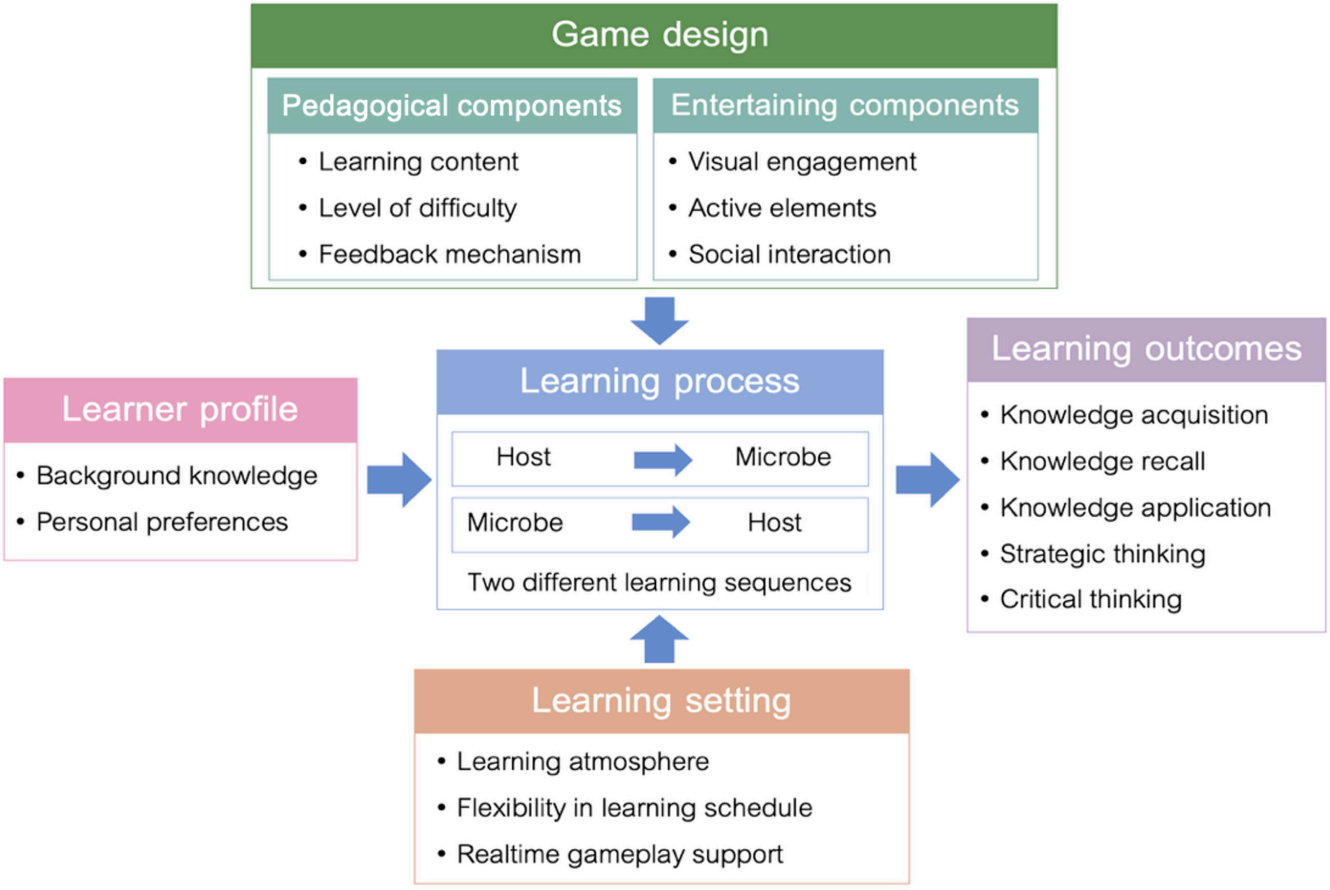
Conceptual framework for the implementation of a dual-role duel card game.

This conceptual framework explains how the emerging themes show the connections between the core elements of game design, learner characteristics, and the learning setting in supporting students to achieve expected learning outcomes (Figure 4). The game should be designed with a balance between pedagogical and entertaining components, supporting students in understanding key concepts while maintaining their motivation throughout the learning process. The learning process is at the center of the framework, where students move between playing as the host and the microbe. By switching roles, students encounter different kinds of challenges, which encourages them to think in new ways and gradually build their knowledge through the game. The game’s rules allow for repeated practice and reflection, helping students remember what they learn and apply it in various situations. The framework highlights the importance of learners’ background knowledge and personal preferences, as well as the influence of the learning settings. Factors such as the learning atmosphere, flexible scheduling, and support during gameplay all play a role in shaping the learning experience. These elements work together with the game design to affect how students participate, interact with each other, and respond to challenges in the game. When these factors are well aligned, the game not only helps students understand the subject content but also supports critical thinking and strategic decision-making skills.

## Discussion

### Effectiveness of Game-Based Learning on Knowledge Enhancement

The “Invasion” game demonstrated its potential as a supplementary learning tool for enhancing knowledge in oral microbiology and immunology. The progressive improvement in scores from Pretest to Posttest 1, and subsequently to Posttest 2, suggests that repeated gameplay contributed to knowledge enhancement. As students moved through the rounds, they were exposed to more diverse sets of microbes, with some organisms appearing more than once. This repetition likely reinforced recall, aligning with established principles of spaced learning and retrieval practice [29]. Participants reported varied learning experiences; some were able to recall prior coursework, others acquired new information, particularly related to antimicrobial treatments, and many developed a broader understanding of how microbiological knowledge applies within clinical contexts. The game was reported by participants to encourage strategic thinking and decision-making skills, which are important for developing clinical reasoning [30-32]. Participants perceived that the dual-role structure of the game, alternating between structured observation and active problem-solving, provided different learning experiences. Students learned not only through success but also through failure perspectives [33,34]. Lost heart points from incorrect guesses can be considered as immediate feedback, prompting them to reflect and adjust their approach. This process reflects the role of failure theory, where knowledge is built through doing, failing, and refining strategies. These findings align with previous research [21,23,28], showing that game-based learning can enhance understanding through engagement, feedback, and active participation.

### Impact of Role Sequence on Cognitive Processing

Although knowledge improvement between groups did not differ significantly, qualitative findings suggest that the sequence in which students engaged with gameplay roles experienced the learning process differently. Students who began as the microbe interacted with the material in a more observational manner, generating clues and observing how their partner responded. This role offered an opportunity to reflect on microbial characteristics without the immediate demand for decision-making. This process aligns with inductive learning, where knowledge is built from observed patterns and structured clues. In contrast, those who started as the host were required to analyze clues and make diagnostic judgments from the beginning, enhancing deeper cognitive processing [35,36]. This approach reflects deductive reasoning, in which learners begin with a hypothesis and test it through gameplay. The effectiveness of each sequence may depend on individual learner traits such as cognitive readiness, prior knowledge, or preferred learning approach [37,38]. While some students appeared to benefit from first observing how the content functioned in context, others were more engaged when challenged to act at an early stage. Across both pathways, students reported cognitive improvement through the interactive and engaging environment, highlighting the importance of role variation in supporting diverse learning styles.

### Influence of Learner Diversity on Gameplay Experiences

In addition to role assignment, other factors also influenced how students experienced the game and developed cognitive skills through play. Background knowledge and prior experience of learners appear to be important factors in shaping their engagement with the game [28]. Those with a stronger background knowledge tended to participate more confidently throughout the activity [39]. It is possible that students who felt less familiar with the content were more likely to prefer the microbe role, where they could rely more on the structured clues provided by the cards. In addition, individual learning preferences can shape students’ approach to gameplay [40-42]; some preferred roles that felt less cognitively demanding, while others were more engaged by strategic decision-making and competitive challenge. The learning context, including peer interaction and minimal technical barriers, can further support student engagement with the game tasks [20,21]. These findings highlight the importance of designing educational games that incorporate role variation and provide flexibility to support learners across a range of backgrounds and preferences.

### Pedagogical Implications and Curriculum Integration

The “Invasion” game was developed to align with the pedagogical goals of dental education by accommodating diverse learning preferences and cognitive styles. Its dual-role format allows students to alternate between reflective observation as the microbe and active problem-solving as the host, supporting both inductive and deductive reasoning [43,44]. This structured variation enables learners to engage with microbiology and immunology content through multiple perspectives, promoting deeper understanding and flexible thinking. Within the dental curriculum, the “Invasion” game can be considered a supplemental learning tool alongside didactic lectures to reinforce basic biomedical knowledge, similar to other game-based strategies successfully applied in preclinical dental education [21]. This is consistent with the argument that game-based learning should not be implemented as a mandatory learning method for learners [45]. The adaptable structure of the “Invasion” game allows flexible integration into tutorials, self-directed study, or flipped classrooms, while its emphasis on peer interaction and strategic decision-making fosters motivation, collaboration, and active learning. Beyond dental education, the dual-role format could also be applied in other health education contexts, such as medical, nursing, and pharmacy curricula, where collaborative learning and clinical decision-making are equally essential, although the present content is more directly aligned with oral microbiology and immunology.

### Limitations and Recommendations for Future Research

While this mixed methods study provided a comprehensive integration of quantitative and qualitative insights into student learning, certain limitations should be acknowledged. The study was conducted at a single institution with a relatively small sample size, which may limit the generalizability of the findings to broader educational contexts. While the test items were randomized in sequence, the repeated use of the same items across all assessment points may have introduced a memory effect, enhancing posttest performance. Future studies could minimize this issue by using different but equivalent test items for pre- and postassessments, supported by analyses of item difficulty and parallel forms reliability. The short duration of the intervention did not allow for evaluation of long-term retention or application to real-world clinical tasks. Future research should incorporate diverse settings, larger cohorts, varied assessment formats, multiple gameplay sessions over time, and delayed postknowledge assessments to better evaluate the long-term educational impact of serious games.

### Conclusions

This study demonstrated that a dual-role educational card game can effectively support diverse learning preferences in oral microbiology and immunology by offering both structured observation and active problem-solving. The game not only enhanced knowledge acquisition but was also perceived by participants to foster strategic thinking and learner engagement through its dynamic role-based format. By alternating between the roles of microbe and host, students engaged with content in different ways, enabling both inductive and deductive learning processes. These findings highlight the value of incorporating flexible, learner-centered tools into the dental curriculum to bridge theoretical science and clinical reasoning. Future research is recommended to investigate the adaptability of this game in broader health education contexts and to evaluate long-term knowledge retention through repeated gameplay sessions over time and follow-up assessments.

## Abbreviations

IOC: The Index of Item-Objective Congruence
